# Type 2 myocardial infarction in a patient with acute abdomen due to an incarcerated Amyand’s Hernia

**DOI:** 10.1016/j.ijscr.2020.03.027

**Published:** 2020-04-01

**Authors:** Paulo Cabrera, Carlos Roman, Silvia Barbosa, Fabian Alvarado, Esteban Diaz, Mayerlin Martinez

**Affiliations:** Fundación Cardioinfantil, General Surgery Department, Bogota, Colombia

**Keywords:** Myocardial infarction, Amyand’s Hernia, Acute abdomen, Type 2 myocardial infarction, Mortality

## Abstract

**Background:**

Type 2 myocardial infarction (MIT2) is characterized by higher mortality rates compared to conventional type 1 infarction according to the European Society of Cardiology (ESC) in 2018. The purpose of this case is to identify appropriate therapeutic measures. A case of an Amyand’s Hernia that produced an MIT2 is described in this work.

**Case report:**

A 77-year-old male was admitted to our emergency department for acute abdominal pain in the right lower quadrant associated with the presence of an ipsilateral inguinal hernia with signs of peritoneal irritation, while complaining of chest pain. A positive troponin indicated the presence of myocardial infarction. A laparotomy was performed with the finding of an incarcerated right inguino-scrotal hernia that contained the gangrenous and perforated cecal appendix (Amyand hernia type 3). The treatment consisted of surgical correction of the hernia, an appendectomy, antibiotics and support in the intensive care unit with a positive outcome. The diagnosis of Amyand hernia type 3 was established intraoperatively, and by imaging, confirming the presence of an MIT2 according to the criteria of the fourth definition of ECS infarction.

**Conclusion:**

In the surgical environment it is strange to find patients who present with acute abdominal pain and a myocardial infarction at the same time. It is necessary for the consultant to recognize these two entities to make a correct diagnosis and provide timely treatment to reduce any possibility of patient mortality.

## Highlights

• The type 2 myocardial infarction is an imbalance in the supply of oxygen to myocardium not related by an atherothrombotic disease which can be caused by an acute abdomen.
• The Amyand’s Hernia with an myocardial ischemic pathology is notoriously uncommon in the surgical setting being of high mortality for the patient.
• Although the holistic approach of patients with acute abdomen is the work of the surgeon, is important the fact of knowing the different patterns of clinical presentation of pathologies that not corresponding to the surgical specialty.

## 1 Introduction

Currently, cardiovascular diseases take more lives than all types of cancer. They are the leading cause of mortality worldwide. In 2013 there were more than 17.3 million deaths, with this statistical number potentially increasing to more than 23.6 million by 2030 [1]. The myocardial infarction (MI) is a cardiovascular disease that currently includes a clinical classification in five types of which two are mentioned here; MIT1 corresponds to an ischemic lesion due to an atherothrombotic coronary disease, and MIT2 represents an ischemic myocardial lesion due to a mismatch between oxygen supply and demand, it follows that acute alteration of the atherothrombotic plaque is not a characteristic of MIT2. MIT1 and MIT2 are the most common MI subtypes, which comprise 98% of all heart attacks and both demand different routes of therapeutic method. While for MIT1 the management includes revascularization and early administration of thrombolytic agents, for MIT2 the focus is on addressing the underlying etiology and correcting the factors that have led to the imbalance between the supply and demand of oxygen [2,4].

Any case of an MIT2 describing a patient with an acute abdomen triggered by an Amyand hernia is rare in the surgical environment with an incidence of less than 1% in all inguinal hernias, and becomes exceptionally rare, with an incidence 0.1%, when complications such as inflammation, perforation or abscess inflammation arise [3]. This case report has been structured according to the international SCARE guidelines [15].

*What procedure should a surgeon take when faced with a patient showing inguinal hernia incarcerated, acute abdomen and symptoms of myocardial infarction?*

## 2 Case report

A 77-year-old male patient with a history of chronic obstructive pulmonary disease was referred to our emergency department as a heart attack case. He presented 4 days of progressively intense pain in the right lower quadrant along with a syncopal episode preceded by dyspnea and oppressive thoracic pain. In the remission site troponin was positive and the electrocardiogram (ECG) was normal; This required initial anti ischemic management and transfer to our service. Upon admission his abdomen showed marked abdominal distension with peritoneal irritation signs, absent bowel sounds and an incarcerated hernia in this level. The patient did not report thoracic pain or dyspnea during the physical exam and the cardiopulmonary auscultation was normal.

**Fig. 1:**
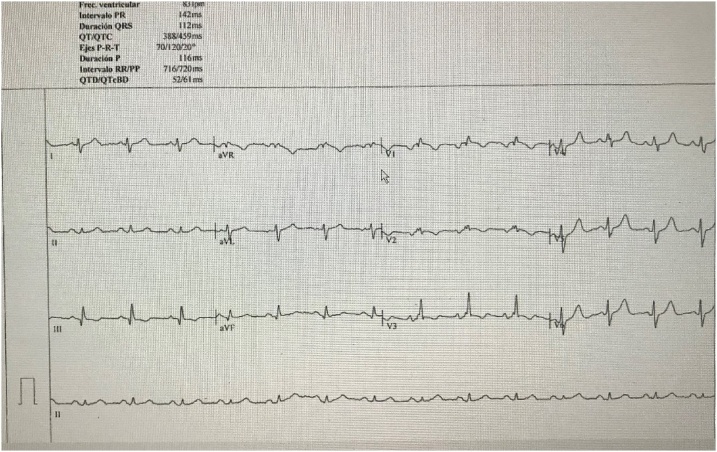
The ECG with Q waves in DII, DIII and AVF, negative T waves in VI V2 and V3 without alteration of the ST segment.

The ECG (Fig. 1) showed Q waves in DII, DIII and AVF, negative T waves in VI V2 and V3 without alteration of the ST segment. The laboratories showed an ultrasensitive positive troponin of 0.040 with a delta positive at 0.030, The blood count, renal function, electrolytes and arterial gases were normal.

**Fig. 2:**
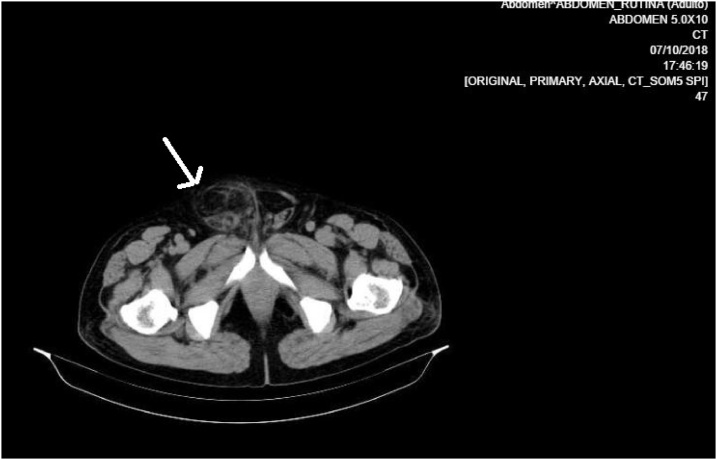
The CT scan showed the Amyand’s hernia.

A transthoracic echocardiogram showed a moderate dilatation of the right ventricle with signs of pressure and pulmonary hypertension without contractility disorders. The complementary computed tomography (CT) showed findings of acute appendicitis and probable Amyand’s Hernia (Fig. 2).

**Fig. 3:**
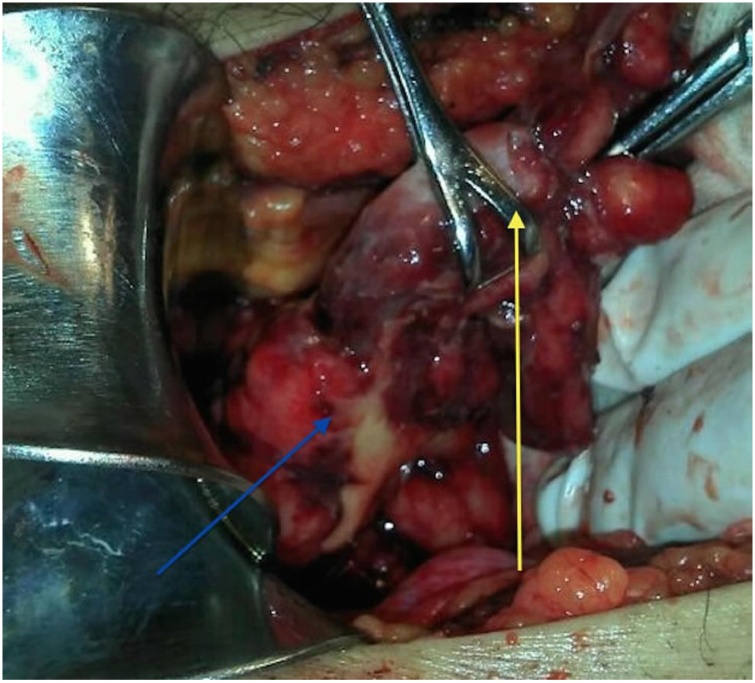
Perforation of the appendix (right arrow; Perforation, left arrow: Hernia sac). Image taken from ELSEVIER, Amyand hernia and complicated appendicitis, Case Report and surgical treatment. E. García J. Martínez, C. Rosales. Surgery and surgeons. 2016; 84 (1): 54–57.

**Fig. 4:**
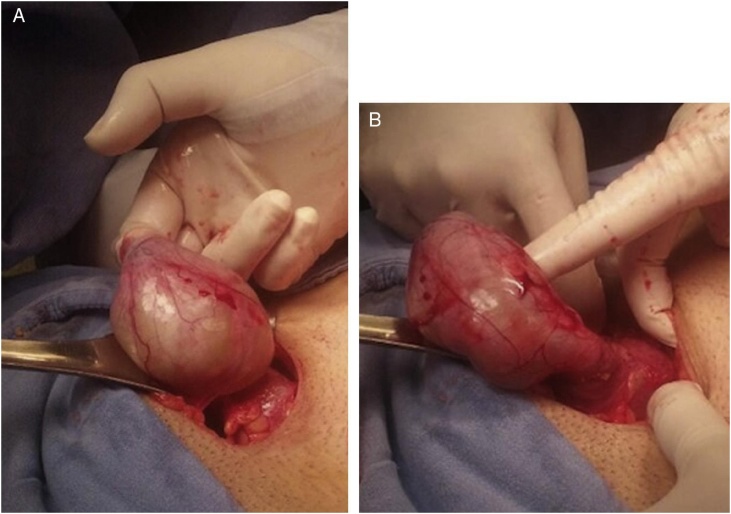
A and B: Presence of the cecal appendix inside the hernia sac. Image taken from: ELSEVIER, Amyand hernia and complicated appendicitis, Case Report and surgical treatment. E. García J. Martínez, C. Rosales. Surgery and surgeons. 2016; 84 (1): 54–57.

The treatment consisted of oral restriction, antibiotics, analgesia, and hydration. This combination was highly approved by the cardiology and surgical teams who decided to perform an emergency laparotomy. The intraoperative findings included a right inguino-scrotal hernia incarcerated with a necrotized hernia sac that contained the cecal appendix which was gangrenous and perforated, with significant tissue edema in the right inguinal region, blind, parietal peritoneum with pelvic peritonitis (Fig. 3, Fig. 4). The patient required Intensive Care with invasive ventilatory support and vasopressor.

With managed multidisciplinary care by intensivists, cardiologists, nutritional support and surgeons, the patient presented an evolution of improvement, achieving, on the second postoperative day, removal of the ventilatory and vasopressor support. He was transferred to the hospitalization area and administered a liquid diet, analgesia and cardiac catheterization to establish the etiology of the MI. Acetylsalicylic acid which was added at a dose of 100 mg every day, along with an angiotensin receptor antagonist (ARA II), due to high blood pressure during hospitalization, and anticoagulation with enoxaparin. He was taken for cardiac catheterization with evidence of healthy coronaries and no signs of atherothrombosis which could lead to the diagnosis of MIT2.

His hospitalization lasted about 6 days with a positive outcome. Consequently, he was discharged with antibiotics therapy for 7 days, analgesia as needed and an outpatient’s order controlled by general surgery and cardiology at 10 days presenting an adequate postoperative.

## 3 Discussion

Myocardial infarction (MI) is part of the cardiovascular diseases that are the leading cause of mortality worldwide [1]. The identification of each of its classifications is important because each entity requires a different treatment according to its cause.

In the surgical field there are pathologies that are discovered only intraoperatively. For this reason, a comprehensive medical history, thorough physical examination of the patient and the complementary exams like a CT scan, can guide us to establish an accurate diagnosis.

The answer regarding the approach to take in a patient diagnosed with MI with signs of acute abdomen is a challenge for any surgeon.

The concept of MI according to the new guidelines of the European Society of Cardiology in 2018 named the fourth definition, tells us that in the diagnosis of acute myocardial infarction cases the indicators are: the increase or fall of markers like troponin, typical symptoms of discomfort in the chest, ischemic changes in the electrocardiogram (ECG) and images on the echocardiogram with a new viable myocardial loss or abnormalities of cardiac wall movements [4].

The MI includes a clinical classification for five types where the most common are; Type 1 myocardial infarction (MIT1) which is defined as necrosis caused by an acute coronary event secondary to the rupture of an atherosclerotic plaque. On the other hand, type 2 myocardial infarction (MIT2) is explained as a myocardial injury caused by an imbalance between the supply and demand of oxygen that occurs between 16% and 71% of cases of MI, the alteration of an atherothrombotic plaque is not a characteristic of this infarction type and the treatment will be the correction of imbalance inducing triggers like severe infections, sepsis and shock in critically ill patients [2].

It is currently known that in MIT2 the long term mortality rates for patients are higher than for MIT1. More than two thirds of patients admitted with MIT2 die in less than 5 years, with the majority, non-cardiovascular deaths [5]. In this case the importance lies in correcting the cause that produced MIT2.

In our case report, the current criteria of the European Society of Cardiology of 2018 were taken into account. It is suggested that the patient with an MIT2 follow a course of diagnostic criteria to establish the increase in troponin values, chest discomfort and evidence of an imbalance in the supply of oxygen to myocardium not related by an atherothrombotic disease, that may have been discarded in an echocardiogram and cardiac catheterization.

With regard to the pathophysiological mechanisms that led to the oxygen imbalance, it was found that it was an Amyand's hernia that was responsible for MIT2 in the patient, through extraluminal obstruction of the appendix. The increase of pressure in the herniated neck limited blood flow resulting in inflammation and bacterial proliferation causing a state of sepsis [6,7]. The diagnosis of Amyand's hernia was based on the contrasted tomography of the abdomen which is the most highly favored diagnostic method at the preoperative level ultimately informing the appropriate surgical repair procedure [8].

The intraoperative surgical treatment of Amyand hernia was based on the classification proposed by Losanoff and Basson; this consists of Amyand hernia type 1 where the cecal appendix is ​​normal, type 2 where the cecal appendix presents some degree of acute appendicitis, type 3 where the appendix presents acute appendicitis and abdominal sepsis or perforation like our patient, and finally the type 4 where the cecal appendix exhibits ​​acute appendicitis with other diseases of abdominal origin not associated [9,10].

**Fig. 5:**
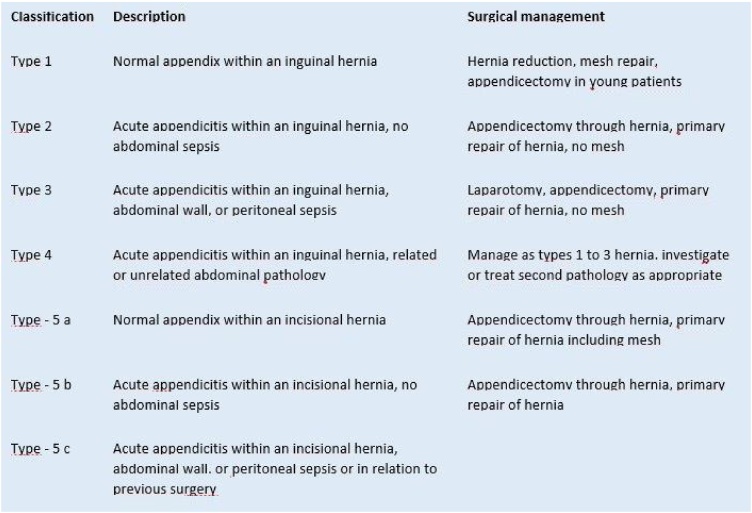
Classification of Amyand Hernias, after Losanoff and Basson, modified by Rikki as Rikki's classification of Amyand Hernias. Image taken from: Rikki S, Gupta S. “Amyand’s Hernia” – Pathophysiology, Role of Investigations and Treatment. Maedica(Buchar). 2011 Oct; 6(4): 321–327.

An appendectomy was necessary, followed by primary repair of the hernia without mesh and a medium laparotomy based on Amyand hernia type 3 according to the classification of Losanoff and Basson. Suffice to say, the treatment of Amyand type 1 hernias is controversial and consists of employing appendectomy procedures. The use of a mesh is contraindicated in cases of obvious infection or peritonitis because it can increase inflammation or overinfect. The classification of Losanoff and Basson was modified by Rikki, et al., who added a fifth type (Fig. 5) [11,12,14].

The mortality of Amyand’s hernia with the perforated appendix ranges from between 15 and 30%; this added to the high mortality stats of MIT2 necessitates the surgeon to be knowledgeable in the pathophysiological mechanisms of these two entities in order to make an adequate diagnosis and give timely treatment saving the patient’s life [13].

## 4 Conclusion

The occurrence of an acute abdomen caused by an Amyand’s hernia with a myocardial ischemic pathology is notoriously uncommon in the surgical setting. However, it is vitally important that the surgeon is knowledgeable regarding these two entities taking into account their high mortality outcomes. Making an adequate diagnosis based on the current scientific literature, can help provide appropriate and timely treatment guidance. Nonetheless, the holistic approach of patients with acute abdomen is largely the work of the surgeon, yet in these cases, knowledge of the different patterns of clinical presentation of pathologies, which do not necessarily correspond to the surgical specialty, require integral care management experts in patient care.

## Declaration of Competing Interest

The authors have no financial or proprietary interest in the subject matter, we do not have any conflict of interest.

## Funding

There was financing by Fundacion Cardioinfantil Bogota Colombia.

## Ethical approval

This article is exempt from ethical approval. Prior informed consent from the patient was obtained for this case report.

## Consent

Written informed consent was obtained from the patient for the publication of this case report and accompanying images which is attached.

## Author contribution

Author 1: Paulo Cabrera; Patient’s clinical history review, informed consent signature, literature review. Article build up and writing, conceptualization, investigation and visualization.

Author 2: Carlos Roman; Article review. Patient’s surgical procedure and clinical follow-up during his hospital stay and ambulatory monitoring, writing, Review, editing and supervision.

Author 3: Silvia Barbosa; Article review. Patient’s clinical history review, literature review. Article build up and writing, investigation and visualization.

Author 4: Andres Alvarado; Article review and build up, editing and supervision.

Author 5: Esteban Diaz; Patient’s clinical history review, literature review and redaction.

Author 5: Mayerlin Martinez; editing, English translation, visualization, literature review.

## Registration of research studies

Does not apply.

## Guarantor

Author 1: Paulo Cabrera.

Author 2: Carlos Roman.

Author 3: Silvia Barbosa.

Author 4: Andres Alvarado.

Author 5: Esteban Diaz.

Author 5: Mayerlin Martinez.

## Provenance and peer review

Editorially reviewed, not externally peer-reviewed.
